# Association between alcohol intake and death from cardiovascular diseases and its subtypes stratified by dyslipidemia in Japanese men: 20-years follow-up of NIPPON DATA90

**DOI:** 10.1265/ehpm.24-00164

**Published:** 2024-11-02

**Authors:** Takumi Hirata, Aya Hirata, Sayuki Torii, Naoyuki Takashima, Aya Kadota, Sohel Reza Choudhury, Akira Okayama, Katsuyuki Miura, Tomonori Okamura, NIPPON DATA90 Study Group

**Affiliations:** 1 Human Care Research Team, Tokyo Metropolitan Institute for Geriatrics and Gerontology, Tokyo, Japan; 2 Department of Preventive Medicine and Public Health, Keio University School of Medicine, Tokyo, Japan; 3 Department of Public Health, Shiga University of Medical Science, Otsu, Japan; 4 Department of Epidemiology for Community Health and Medicine, Kyoto Prefectural University of Medicine, Kyoto, Japan; 5 NCD Epidemiology Research Center, Shiga University of Medical Science, Otsu, Japan; 6 Department of Epidemiology and Research, National Heart Foundation Hospital and Research Institute, Dhaka, Bangladesh; 7 The Research Institute of Strategy for Prevention, Tokyo, Japan

**Keywords:** Alcohol intake, Cardiovascular disease, Cohort study, Dyslipidemia

## Abstract

**Background:**

The association between alcohol consumption and atherosclerotic cardiovascular disease (ASCVD) was controversial, and no previous studies have shown the impact of dyslipidemia on the association. We aimed to clarify the association between alcohol consumption and death from cardiovascular disease (CVD) and its subtypes and the impact of dyslipidemia on the association.

**Methods:**

We conducted a 20-year cohort study to clarify the association between alcohol intake and death from CVD and its subtypes in 2,909 Japanese men. We estimated the hazard ratio (HR) and 95% confidence intervals (CIs) for current drinkers with non-drinkers as the reference, after adjusting for potential confounders using Cox proportional hazards models. We also investigated the association between alcohol consumption and ASCVD or CVD death stratified by the presence or absence of dyslipidemia.

**Results:**

During 50,782 person-years of follow-up period, 223 participants died from total CVD, 110 participants died from ASCVD, and 25 participants died from cerebral hemorrhage. Current drinkers with 1 *gou*/day were significantly associated with lower risk of ASCVD (HR: 0.60, 95%CI: 0.37–0.98), which is more apparent in those without dyslipidemia, and current drinkers with ≥3 *gou*/day were significantly associated with higher risk of cerebral hemorrhage (HR: 4.13, 95%CI: 1.12–15.19).

**Conclusions:**

Small amounts of alcohol drinking were associated with lower risk of ASCVD in Japanese men, especially those without dyslipidemia. Meanwhile, excessive alcohol drinking was associated with higher risk of cerebral hemorrhage. Our findings suggest that it is important for current Japanese drinkers to reduce alcohol consumption for preventing ASCVD or cerebral hemorrhage.

**Supplementary information:**

The online version contains supplementary material available at https://doi.org/10.1265/ehpm.24-00164.

## Introduction

Alcohol consumption, especially excessive alcohol drinking, is associated with several health problems including esophageal and gastric cancers, hepatic steatosis, pancreatitis and cardiovascular disease (CVD) [1–4]. Regarding CVD or atherosclerotic cardiovascular disease (ASCVD), small-to-moderate alcohol consumption has been reported to be associated with reduced risk [4, 5]. Several observational studies have shown the association between alcohol consumption and CVD in Japanese population [5–11], and these reports suggested the risk of alcohol consumption on CVD was differed by sex and the subtype of CVD. Most of these studies were conducted on men because the incidence of CVD and the amount of alcohol consumption in women is significantly lower than those in men [6–8]. In Japanese men, heavy alcohol consumption was associated with increased risk of total stroke, in particular hemorrhagic stroke [5, 9, 11], while the association between alcohol consumption and ASCVD was controversial. These previous findings suggest that it is necessary to clarify the association between alcohol consumption and ASCVD using other large-scale database of Japanese men.

Meanwhile, several risk factors such as hypertension, diabetes, and dyslipidemia are expected to influence the association between alcohol consumption and CVD because excessive alcohol consumption is associated with increased risk of hypertension, increased levels of high-density lipoprotein (HDL) cholesterol and decreased levels of low-density lipoprotein (LDL) cholesterol [5, 12–17], and there is a U-shaped association between alcohol consumption and incident diabetes [18]. Actually, a previous Japanese study has reported that the association between alcohol consumption and the incidence of CVD was U-shaped in men with hypertension, but not in those without hypertension [6]. Another study has reported that the reduction in CVD risk with drinking was more pronounced in the non-diabetic population compared to the diabetic population [19]. Meanwhile, there are few studies on the impact of lipids on the association between alcohol consumption and CVD. As mentioned earlier, alcohol consumption increases levels of HDL-C and decreases levels of LDL-C. Although at first glance, this phenomenon appears favorable for the prevention of atherosclerosis, it is undetermined. HDL-C levels are increased by alcohol consumption, meanwhile the effect of alcohol consumption on HDL anti-atherogenic function is controversial [20, 21]. In addition, LDL modification such as oxidation causes atherogenesis, which could be associated with alcohol consumption [22].

Therefore, we expected that examining the impact of dyslipidemia on the association between alcohol consumption and CVD would provide insights that reflect the function and dynamics of lipids as affected by alcohol consumption. It is important to clarify them in the setting of both clinical medicine and public health. Therefore, we aimed to clarify the association between alcohol consumption and death from CVD and its subtypes using data from a large-scale epidemiological study of Japanese men. We also investigated that the impact of dyslipidemia on the association between alcohol consumption and death from CVD and its subtypes in the present study.

## Methods

### Participants

The National Integrated Project for Prospective Observation if Non-communicable Disease And its Trends in the Aged (NIPPON DATA) is a series of cohort studies conducted by the National Survey on Circulatory Disorders of Japan. NIPPON DATA90 was performed from the Fourth National Survey on Circulatory Disorders of Japan in 1990. Detailed methods in constructing the cohort were described elsewhere [23–25]. We analyzed the 20-year follow-up data of NIPPON DATA90 in the present study. The NIPPON DATA90 was approved by the Institutional Review Board of Shiga University of Medical Science (R2005-021).

We recruited a total of 3,504 men who were community residents aged 30 years or older from 300 randomly selected districts across Japan. Among all participants, we excluded 595 men with past history of CVD (n = 185) or unknown past history of CVD (n = 46), missing data on death (n = 98), body mass index (BMI; n = 2), treatment for hypertension, dyslipidemia or diabetes (n = 65), and laboratory data (n = 199). The remaining 2,909 men were included in the present analysis.

### Outcome measurements

The outcome of the present study was death from coronary heart disease (CHD), ischemic stroke, cerebral hemorrhage, ASCVD and total CVD. The cause of death was identified using the National Vital Statistic data. The underlying causes of death in the National Vital Statistics were coded according to the 9^th^ International Classification of Disease (ICD-9) until the end of 1994 and according to the 10^th^ International Classification of Disease (ICD-10) from 1995. Deaths from any CVD were identified by ICD-9 codes (393–459) and ICD-10 codes (I00–I99). CHD was defined with ICD-9 codes 410 to 414 and ICD-10 codes I20 to I25, ischemic stroke was defined with ICD-9 codes 433, 434, 437.8a, and 437.8b and ICD-10 codes I63 and I69.3, and cerebral hemorrhage was defined with ICD-9 codes 431 to 432 and ICD-10 codes I61 and I69.1. ASCVD was defined as CHD and ischemic stroke. The details of the classification are described elsewhere [26–28].

### Exposure measurements

The main exposure of the present study was alcohol drinking status. Public health nurses survey asked the frequency of drinking per week and the average daily alcohol consumption (*gou*) on drinking days. Based on the National Survey on Circulatory Disorders of Japan rules, current drinkers were defined as those who drank three or more times a week and drank one or more drinks per day on drinking days. We classified all participants into the following five groups based on the alcohol drinking status: non-drinker, past drinker, current drinkers G1, current drinkers G2, current drinkers G3, in which current drinkers G1 were defined as those who drank 3 or more days per week and about 1 *gou* of alcohol per day, G2 as those who drank 3 or more days per week and about 2 *gou* of alcohol per day, and G3 as those who drank 3 or more days per week and 3 *gou* or more of alcohol per day. The *gou* is a traditional Japanese alcohol drinking unit, and 1 *gou* is equivalent to 180 mL of sake (Japanese rice wine), which contains 23.0 g of alcohol. Its alcohol content is roughly equivalent to 663 mL (1 bottle) of beer, 70 mL (two single shots) of whisky, or 110 mL (a half glass) of “shochu” (spirits made from barley, sweet potato, rice or any combination of these).

### Covariate measurements

Public health nurses measured data including smoking habit as well as current health status and medical history using questionnaire. All participants were asked about current smoking status (current smoking, past smoking and never-smoking), the number of cigarettes per day and the duration of smoking. Based on these information, smoking habit was categorized into non-current smokers and current smokers in the present study. BMI was calculated as weight (kg) divided by the square of height (m). Blood pressure was measured once by trained observers using a standard mercury sphygmomanometer on the right arm of seated participants, and we defined hypertension as systolic blood pressure (SBP) ≥140 mmHg, diastolic blood pressure (DBP) ≥90 mmHg, or receiving medication for hypertension.

Non-fasting blood samples were obtained at the baseline survey. The serum was separated and centrifuged soon after blood coagulation. Plasma samples were collected in siliconized tubes containing sodium fluoride and shipped to one laboratory (SRL, Tokyo, Japan) for blood measurements. Plasma glucose was measured enzymatically. Glycated hemoglobin (HbA1c; JDS) values were converted to HbA1c (NGSP) values using the formula proved by JDS: HbA1c NGSP value (%) = 1.02 × JDS value (%) + 0.25 [29]. We used HbA1c NGSP values in the present analyses. We defined diabetes as non-fasting blood glucose ≥200 mg/dL, HbA1c (NGSP) ≥6.5%, or taking medicine for diabetes. Serum total cholesterol and serum triglycerides were measured enzymatically, and high-density lipoprotein cholesterol was measured using heparin-calcium precipitation method. Non-HDL cholesterol (non HDL-C) was calculated by subtracting HDL-C from total cholesterol, and we defined dyslipidemia as non HDL-C ≥170 mg/dL, TG ≥150 mg/dL, HDL-C <40 mg/dL, or receiving medication for dyslipidemia. Gamma glutamyl transpeptidase (γ-GTP) was measured using 3-carboxyl-4-nitroanilide substrate method. Alanine aminotransferase (ALT) and aspartate aminotransferase (AST) were measured using ultraviolet methods.

### Statistical analysis

Continuous variables are reported as the mean and standard deviation or the median and interquartile range, and categorical variables are reported as the number and proportion. We calculated the crude mortality rate and the hazard ratio (HR) for death from CHD, ischemic stroke, cerebral hemorrhage, ASCVD and total CVD. We estimated the HR and 95% confidence intervals (CIs) for current drinkers with non-drinkers as the reference, after adjusting for potential confounders using Cox proportional hazards models. Confounding factors included age (continuous variable), current smoking (categorical variable; yes/no), exercise habit (categorical variable; yes/no), BMI (continuous variable), diabetes (categorical variable; yes/no), dyslipidemia (categorical variable; yes/no) and hypertension (categorical variable; yes/no). As an additional statistical analysis, γ-GTP, ALT, and AST were also adjusted in the statistical model. Furthermore, we performed stratified analyses of the association between alcohol drinking status and total CVD mortality and ASCVD mortality by the presence of dyslipidemia, non-HDL-C levels (<170 mg/dL, ≥170 mg/dL), TG levels (<150 mg/dL, ≥150 mg/dL), and HDL-C levels (<40 mg/dL, ≥40 mg/dL), respectively. We also added interaction terms of each type of dyslipidemia and alcohol drinking status (non-drinker, past drinker, current drinkers G1, current drinkers G2, current drinkers G3) to the model adjusted for age, smoking status, exercise habits, BMI, diabetes, and hypertension to examine the effect of dyslipidemia on the association between alcohol drinking status and CVD and ASCVD mortality, respectively. To remove the effect of alcohol drinking cessation due to health problem, the analysis excluding those who deceased within 5 years from the baseline survey was conducted. Besides, the analysis was conducted by including alcohol consumption as a continuous variable in the model, except for past drinkers. All analyses were performed using STATA SE 13 data analysis and statistical software (Stata Corp LP, College Station, TX, USA). All *P* values for statistical tests were two-sided, and *P* < 0.05 was considered statistically significant.

## Results

Clinical characteristics of all participants according to the alcohol drinking status were shown in Table 1. Among all participants, 1,011 participants were non-drinkers, 176 participants were past drinkers, 864 participants were current drinkers G1, 601 participants were current drinkers G2, and 257 participants were current drinkers G3. Mean BMI, the proportion of current smokers, the proportion of participants with hypertension and levels of γ-GTP were higher and mean age was younger with the amount of alcohol consumption.

**Table 1:** Clinical characteristics of all participants according to the alcohol drinking status

	**Non-drinker**	**Past drinker**	**Current drinker**		
			**G1**	**G2**	**G3**
Participants, *n*	1,011	176	864	601	257
Age (years)	53.3 (14.4)	60.0 (14.4)	52.7 (13.2)	50.8 (11.7)	49.3 (10.7)
Body mass index (kg/m^2^)	22.8 (3.1)	22.7 (3.3)	22.9 (2.9)	23.3 (2.9)	23.4 (3.0)
Current smoker, *n* (%)	495 (49.0%)	84 (47.7%)	486 (56.3%)	385 (64.1%)	177 (68.9%)
Exercise habit, *n* (%)	203 (20.1%)	43 (24.4%)	211 (24.4%)	136 (22.6%)	52 (20.2%)
Hypertension, yes, *n* (%)	410 (40.6%)	108 (61.4%)	439 (50.8%)	325 (54.1%)	139 (54.1%)
Systolic blood pressure (mmHg)	134.3 (19.9)	140.5 (21.6)	138.3 (20.1)	139.3 (19.5)	140.7 (19.8)
Diastolic blood pressure (mmHg)	81.2 (11.0)	83.2 (12.7)	84.1 (11.4)	85.8 (11.9)	86.3 (11.7)
Treatment for hypertension, *n* (%)	93 (9.2%)	36 (20.5%)	114 (13.2%)	82 (13.6%)	26 (10.1%)
Diabetes, yes, *n* (%)	57 (5.6%)	24 (13.6%)	51 (5.9%)	26 (4.3%)	15 (5.8%)
Plasma glucose (mg/dL)	103.5 (35.2)	108.6 (46.9)	102.0 (32.4)	102.4 (29.8)	104.7 (42.3)
Hemoglobin A1c (%)	5.3 (0.8)	5.4 (0.9)	5.4 (0.7)	5.4 (0.8)	5.4 (0.8)
Treatment for diabetes, *n* (%)	29 (2.9%)	17 (9.7%)	34 (3.9%)	9 (1.5%)	5 (2.0%)
Dyslipidemia, *n* (%)	578 (57.2%)	107 (60.8%)	419 (48.5%)	282 (46.9%)	132 (51.4%)
Total cholesterol (mg/dL)	199.2 (36.3)	197.3 (45.1)	197.1 (35.2)	198.3 (35.3)	198.5 (39.6)
Triglycerides (mg/dL)	116 (83–171)	117 (81–187)	116 (80–180)	124 (86–186)	140 (91–209)
≥150 mg/dL, *n* (%)	341 (33.7%)	68 (38.6%)	292 (33.8%)	226 (37.6%)	119 (46.3%)
HDL cholesterol (mg/dL)	45.9 (13.3)	45.7 (13.7)	51.8 (14.5)	55.0 (15.2)	55.9 (17.9)
<40 mg/dL, *n* (%)	364 (36.0%)	62 (35.2%)	157 (18.2%)	75 (12.5%)	40 (15.6%)
Non-HDL cholesterol (mg/dL)	153.3 (37.2)	151.6 (44.6)	145.3 (36.8)	143.3 (37.6)	142.6 (43.1)
≥170 mg/dL, *n* (%)	310 (30.7%)	50 (28.4%)	215 (24.9%)	138 (23.0%)	57 (22.2%)
Treatment for dyslipidemia, *n* (%)	22 (2.2%)	10 (5.7%)	13 (1.5%)	8 (1.3%)	5 (2.0%)
γ-GTP (IU/L)	22 (16–35)	26 (19–45)	33 (20–57)	49 (29–88)	62 (37–116)
AST (IU/L)	22 (18–27)	23 (19–30)	24 (20–28)	25 (21–31)	27 (22–34)
ALT (IU/L)	21 (15–32)	22 (15–36)	21 (15–31)	23 (17–34)	25 (19–39)

HDL, High-density lipoprotein; γ-GTP, gamma glutamyl-transpeptidase; ALT, alanine aminotransferase; AST, aspartate aminotransferase

Continuous variable is expressed as mean (standard deviation) or median (interquartile range)

Categorical variable is expressed as number (percentages)

Current drinkers G1 were defined as those who drank 3 or more days per week and about 1 gou of alcohol per day, G2 as those who drank 3 or more days per week and about 2 gou of alcohol per day, and G3 as those who drank 3 or more days per week and 3 gou or more of alcohol per day.

Associations between alcohol drinking status and cause-specific death were shown in Table 2. During 50,782 person-years of follow-up period, 223 participants died from total CVD, 110 participants died from ASCVD (55 participants died from CHD and 55 participants died from ischemic stroke), and 25 participants died from cerebral hemorrhage. The crude mortality rates were 4.39 (95% CI: 3.85–5.00), 2.16 (95% CI: 1.79–2.61), 1.08 (95% CI: 0.83–1.41), 1.08 (95% CI: 0.83–1.41), 0.49 (95% CI: 0.33–0.72) per 1,000 person-years for total CVD, ASCVD, CHD, ischemic stroke and cerebral hemorrhage, respectively. The risk of death from total CVD, ASCVD, CHD, and ischemic stroke was lower in the drinker groups than in the non-drinker group. On the other hand, the risk of death from cerebral hemorrhage was higher in the drinker groups than in the non-drinker group. Current drinkers G1 were significantly associated with lower risk of ASCVD mortality compared with non-drinkers after adjusting for confounders (HR: 0.60, 95% CI: 0.37–0.98). Current drinkers G3 were significantly associated with higher risk of cerebral hemorrhage compared with non-drinkers (HR: 4.13, 95% CI: 1.12–15.19). The risk of past drinkers for total CVD and cause-specific CVD death was relatively higher than non-drinkers.

**Table 2:** Associations between alcohol drinking status and each cause-specific death in men

	**Number of participants**	**Number of deaths**	**Follow-up period (py)**	Crude mortality rate* (95% CI)	**Age-adjusted HR (95% CI)**	Multivariable-adjusted HR** (95% CI)
Total cardiovascular disease						
Non-drinker	1,011	87	17,414	5.0 (4.0–6.2)	Ref.	Ref.
Past drinker	176	28	2,627	10.7 (7.4–15.4)	1.43 (0.93–2.19)	1.33 (0.86–2.04)
Current drinker G1	864	57	15,199	3.8 (2.9–4.9)	0.82 (0.59–1.15)	0.75 (0.53–1.05)
Current drinker G2	601	37	10,913	3.4 (2.5–4.7)	0.94 (0.63–1.38)	0.82 (0.55–1.23)
Current drinker G3	257	14	4,629	3.0 (1.8–5.1)	1.03 (0.59–1.83)	0.85 (0.48–1.52)

Atherosclerotic cardiovascular disease						
Non-drinker	1,011	47	17,414	2.7 (2.0–3.6)	Ref.	Ref.
Past drinker	176	14	2,627	5.3 (3.2–9.0)	1.31 (0.72–2.38)	1.13 (0.62–2.08)
Current drinker G1	864	26	15,199	1.7 (1.2–2.5)	0.69 (0.43–1.12)	0.60 (0.37–0.98)
Current drinker G2	601	18	10,913	1.6 (1.0–2.6)	0.85 (0.49–1.47)	0.72 (0.41–1.27)
Current drinker G3	257	5	4,629	1.1 (0.4–2.6)	0.69 (0.28–1.75)	0.55 (0.22–1.40)

Coronary heart disease						
Non-drinker	1,011	23	17,414	1.3 (0.9–2.0)	Ref.	Ref.
Past drinker	176	6	2,627	2.3 (1.0–5.1)	1.20 (0.49–2.96)	1.08 (0.43–2.68)
Current drinker G1	864	13	15,199	0.9 (0.5–1.5)	0.68 (0.35–1.35)	0.62 (0.31–1.24)
Current drinker G2	601	10	10,913	0.9 (0.5–1.7)	0.87 (0.41–1.84)	0.76 (0.35–1.65)
Current drinker G3	257	3	4,629	0.6 (0.2–2.0)	0.74 (0.22–2.48)	0.60 (0.18–2.04)

Ischemic stroke						
Non-drinker	1,011	24	17,414	1.4 (0.9–2.1)	Ref.	Ref.
Past drinker	176	8	2,627	3.0 (1.5–6.1)	1.40 (0.63–3.14)	1.17 (0.52–2.65)
Current drinker G1	864	13	15,199	0.9 (0.5–1.5)	0.71 (0.36–1.39)	0.58 (0.29–1.16)
Current drinker G2	601	8	10,913	0.7 (0.4–1.5)	0.84 (0.37–1.87)	0.67 (0.29–1.55)
Current drinker G3	257	2	4,629	0.4 (0.1–1.7)	0.62 (0.15–2.64)	0.49 (0.11–2.09)

Cerebral hemorrhage						
Non-drinker	1,011	5	17,414	0.3 (0.1–0.7)	Ref.	Ref.
Past drinker	176	2	2,627	0.8 (0.2–3.0)	2.03 (0.39–10.55)	2.08 (0.40–10.83)
Current drinker G1	864	6	15,199	0.4 (0.2–0.9)	1.41 (0.43–4.62)	1.25 (0.38–4.17)
Current drinker G2	601	7	10,913	0.6 (0.3–1.3)	2.61 (0.82–8.27)	2.11 (0.64–6.91)
Current drinker G3	257	5	4,629	1.1 (0.4–2.6)	5.08 (1.45–17.85)	4.13 (1.12–15.19)

py, person-years; HR, hazard ratio; CI, confidence interval

*per 1,000 py

**Adjusted for age, body mass index, diabetes, hypertension, current smoking, exercise habit and dyslipidemia

Current drinkers G1 were defined as those who drank 3 or more days per week and about 1 gou of alcohol per day, G2 as those who drank 3 or more days per week and about 2 gou of alcohol per day, and G3 as those who drank 3 or more days per week and 3 gou or more of alcohol per day.

Associations between alcohol drinking status and death from total CVD or ASCVD stratified by the presence of each type of dyslipidemia were shown in Table 3 (total CVD) and Table 4 (ASCVD). The risk of past drinkers for total CVD and ASCVD deaths was relatively higher than that of non-drinkers in individuals with each type of dyslipidemia, but not in any type of dyslipidemia (*p* for interaction of dyslipidemia and past drinkers for CVD death and ASCVD death: 0.029 and 0.048, respectively). The risk of current drinkers for CVD death was higher than that of non-drinkers in the high non-HDL-C population, but not in the low non-HDL-C population (*p* for interaction of high non-HDL-C and current drinkers G1, G2, and G3: 0.038, 0.246, and 0.151, respectively). The risk of current drinkers for ASCVD death was lower than that of non-drinkers in the high HDL-C population, but not in the low HDL-C population (*p* for interaction of low HDL-C and current drinkers G1, G2, and G3 and high HDL-C: 0.072, 0.367, and 0.459, respectively). The trend of the results remained unchanged when adjusting for liver function test items such as γ-GTP, ALT, and AST, or when excluding those who deceased within 5 years from the baseline survey as the additional analysis (data not shown). The result of associations between alcohol consumption as continuous variable and cardiovascular disease death was presented in Supplemental Table 1 and Supplemental Table 2.

**Table 3:** Associations between alcohol intake and total CVD death according to dyslipidemia in men

	**Number of participants**	**Number of deaths**	**Follow-up period (py)**	Crude mortality rate* (95% CI)	**Age-adjusted HR (95% CI)**	Multivariable-adjusted HR** (95% CI)	***p* for interaction**
With dyslipidemia (*n* = 1,518)							
Non-drinker	578	41	10,136	4.0 (3.0–5.5)	Ref.	Ref.	
Past drinker	107	21	1,660	12.6 (8.2–19.4)	2.21 (1.30–3.75)	1.99 (1.17–3.39)	0.029
Current drinker G1	419	26	7,507	3.5 (2.4–5.1)	0.93 (0.57–1.52)	0.94 (0.57–1.55)	0.204
Current drinker G2	282	14	5,173	2.7 (1.6–4.6)	0.98 (0.53–1.82)	0.96 (0.51–1.78)	0.434
Current drinker G3	132	9	2,357	3.8 (2.0–7.3)	1.32 (0.64–2.71)	1.28 (0.62–2.67)	0.194
Without dyslipidemia (*n* = 1,391)							
Non-drinker	433	46	7,278	6.3 (4.7–8.4)	Ref.	Ref.	
Past drinker	69	7	967	7.2 (3.5–15.2)	0.70 (0.32–1.55)	0.64 (0.29–1.43)	
Current drinker G1	445	31	7,691	4.0 (2.8–5.7)	0.73 (0.46–1.15)	0.58 (0.36–0.92)	
Current drinker G2	319	23	5,739	4.0 (2.7–6.0)	0.88 (0.53–1.46)	0.64 (0.38–1.09)	
Current drinker G3	125	5	2,272	2.2 (0.9–5.3)	0.81 (0.32–2.08)	0.53 (0.20–1.39)	

Higher non-HDL-C (*n* = 770; ≥170 mg/dL)							
Non-drinker	310	19	5,439	3.5 (2.2–5.5)	Ref.	Ref.	
Past drinker	50	11	801	13.7 (7.6–24.8)	3.18 (1.51–6.70)	3.00 (1.41–6.41)	0.027
Current drinker G1	215	16	3,961	4.0 (2.5–6.6)	1.52 (0.78–2.98)	1.43 (0.73–2.83)	0.038
Current drinker G2	138	8	2,563	3.1 (1.6–6.2)	1.43 (0.62–3.32)	1.34 (0.57–3.16)	0.246
Current drinker G3	57	4	1,065	3.8 (1.4–10.0)	1.90 (0.64–5.64)	1.90 (0.62–5.80)	0.151
Lower non-HDL-C (*n* = 2,139; <170 mg/dL)							
Non-drinker	701	68	11,975	5.7 (4.5–7.2)	Ref.	Ref.	
Past drinker	126	17	1,826	9.3 (5.8–15.0)	1.03 (0.60–1.75)	0.95 (0.56–1.63)	
Current drinker G1	649	41	11,238	3.6 (2.7–5.0)	0.68 (0.46–0.999)	0.60 (0.41–0.90)	
Current drinker G2	463	29	8,350	3.5 (2.4–5.0)	0.83 (0.54–1.29)	0.71 (0.45–1.12)	
Current drinker G3	200	10	3,564	2.8 (1.5–5.2)	0.86 (0.44–1.68)	0.67 (0.34–1.32)	

Higher triglycerides (*n* = 1,046; ≥150 mg/dL)							
Non-drinker	341	25	6,071	4.1 (2.8–6.1)	Ref.	Ref.	
Past drinker	68	11	1,106	9.9 (5.5–18.0)	1.85 (0.91–3.77)	1.54 (0.75–3.17)	0.643
Current drinker G1	292	12	5,323	2.3 (1.3–4.0)	0.57 (0.28–1.13)	0.54 (0.27–1.07)	0.310
Current drinker G2	226	13	4,146	3.1 (1.8–5.4)	1.08 (0.55–2.12)	0.97 (0.48–1.94)	0.414
Current drinker G3	119	9	2,119	4.2 (2.2–8.2)	1.41 (0.66–3.04)	1.24 (0.56–2.72)	0.247
Lower triglycerides (*n* = 1,863; <150 mg/dL)							
Non-drinker	670	62	11,343	5.5 (4.3–7.0)	Ref.	Ref.	
Past drinker	108	17	1,521	11.2 (6.9–18.0)	1.24 (0.72–2.12)	1.20 (0.70–2.06)	
Current drinker G1	572	45	9,876	4.6 (3.4–6.1)	0.94 (0.64–1.38)	0.84 (0.57–1.24)	
Current drinker G2	375	24	6,767	3.5 (2.4–5.3)	0.87 (0.54–1.40)	0.75 (0.46–1.21)	
Current drinker G3	138	5	2,510	2.0 (0.8–4.8)	0.78 (0.31–1.95)	0.62 (0.24–1.56)	

Lower HDL-C (*n* = 698; <40 mg/dL)							
Non-drinker	364	28	6,325	4.4 (3.1–6.4)	Ref.	Ref.	
Past drinker	62	13	944	13.8 (8.0–23.7)	1.97 (1.01–3.83)	1.88 (0.95–3.69)	0.219
Current drinker G1	157	15	2,754	5.4 (3.3–9.0)	1.26 (0.68–2.37)	1.24 (0.66–2.33)	0.077
Current drinker G2	75	3	1,370	2.2 (0.7–6.8)	0.77 (0.23–2.54)	0.76 (0.23–2.56)	0.939
Current drinker G3	40	1	706	1.4 (0.2–10.1)	0.48 (0.07–3.55)	0.47 (0.06–3.45)	0.566
Higher HDL-C (*n* = 2,211; ≥40 mg/dL)							
Non-drinker	647	59	11,090	5.3 (4.1–6.9)	Ref.	Ref.	
Past drinker	114	15	1,683	8.9 (5.4–14.8)	1.19 (0.67–2.09)	1.03 (0.58–1.83)	
Current drinker G1	707	42	12,444	3.4 (2.5–4.6)	0.73 (0.49–1.08)	0.61 (0.41–0.92)	
Current drinker G2	526	34	9,543	3.6 (2.5–5.0)	0.95 (0.62–1.45)	0.75 (0.48–1.17)	
Current drinker G3	217	13	3,923	3.3 (1.9–5.7)	1.15 (0.62–2.10)	0.85 (0.45–1.58)	

CVD, cardiovascular disease; HDL-C, high-density lipoprotein cholesterol; py, person-years; HR, hazard ratio; CI, confidence interval

*per 1,000 py

**Adjusted for age, body mass index, diabetes, hypertension, current smoking, and exercise habit

Current drinkers G1 were defined as those who drank 3 or more days per week and about 1 gou of alcohol per day, G2 as those who drank 3 or more days per week and about 2 gou of alcohol per day, and G3 as those who drank 3 or more days per week and 3 gou or more of alcohol per day.

**Table 4:** Associations between alcohol intake and ASCVD death according to dyslipidemia in men

	**Number of participants**	**Number of deaths**	**Follow-up period (py)**	Crude mortality rate* (95% CI)	**Age-adjusted HR (95% CI)**	Multivariable-adjusted HR** (95% CI)	***p* for interaction**
With dyslipidemia (*n* = 1,518)							
Non-drinker	578	21	10,136	2.1 (1.4–3.2)	Ref.	Ref.	
Past drinker	107	11	1,660	6.6 (3.7–12.0)	2.23 (1.07–4.64)	1.90 (0.90–4.00)	0.048
Current drinker G1	419	12	7,507	1.6 (0.9–2.8)	0.85 (0.42–1.72)	0.84 (0.41–1.71)	0.240
Current drinker G2	282	9	5,173	1.7 (0.9–3.3)	1.28 (0.58–2.81)	1.26 (0.56–2.82)	0.064
Current drinker G3	132	2	2,357	0.8 (0.2–3.4)	0.56 (0.13–2.41)	0.56 (0.13–2.41)	0.913
Without dyslipidemia (*n* = 1,391)							
Non-drinker	433	26	7,278	3.6 (2.4–5.2)	Ref.	Ref.	
Past drinker	69	3	967	3.1 (1.0–9.6)	0.52 (0.16–1.73)	0.44 (0.13–1.47)	
Current drinker G1	445	14	7,691	1.8 (1.1–3.1)	0.58 (0.30–1.11)	0.43 (0.22–0.85)	
Current drinker G2	319	9	5,739	1.6 (0.8–3.0)	0.61 (0.28–1.32)	0.40 (0.18–0.89)	
Current drinker G3	125	3	2,272	1.3 (0.4–4.1)	0.91 (0.27–3.12)	0.55 (0.16–1.92)	

Higher non-HDL-C (*n* = 770; ≥170 mg/dL)							
Non-drinker	310	13	5,439	2.4 (1.4–4.1)	Ref.	Ref.	
Past drinker	50	4	801	5.0 (1.9–13.3)	1.69 (0.55–5.19)	1.59 (0.51–5.01)	0.674
Current drinker G1	215	8	3,961	2.0 (1.0–4.0)	1.09 (0.45–2.66)	1.01 (0.41–2.48)	0.241
Current drinker G2	138	4	2,563	1.6 (0.6–4.2)	1.07 (0.34–3.35)	1.02 (0.32–3.27)	0.623
Current drinker G3	57	1	1,065	0.9 (0.1–6.7)	0.69 (0.09–5.29)	0.67 (0.08–5.35)	0.906
Lower non-HDL-C (*n* = 2,139; <170 mg/dL)							
Non-drinker	701	34	11,975	2.8 (2.0–4.0)	Ref.	Ref.	
Past drinker	126	10	1,826	5.5 (2.9–10.2)	1.18 (0.58–2.41)	1.03 (0.50–2.12)	
Current drinker G1	649	18	11,238	1.6 (1.0–2.5)	0.60 (0.34–1.06)	0.52 (0.29–0.92)	
Current drinker G2	463	14	8,350	1.7 (1.0–2.8)	0.81 (0.43–1.52)	0.67 (0.35–1.28)	
Current drinker G3	200	4	3,564	1.1 (0.4–3.0)	0.71 (0.25–2.02)	0.52 (0.18–1.49)	

Higher triglycerides (*n* = 1,046; ≥150 mg/dL)							
Non-drinker	341	13	6,071	2.1 (1.2–3.7)	Ref.	Ref.	
Past drinker	68	6	1,106	5.4 (2.4–12.1)	1.90 (0.72–5.03)	1.41 (0.52–3.81)	0.564
Current drinker G1	292	5	5,323	0.9 (0.4–2.3)	0.46 (0.17–1.30)	0.42 (0.15–1.20)	0.408
Current drinker G2	226	8	4,146	1.9 (1.0–3.9)	1.37 (0.56–3.35)	1.25 (0.49–3.15)	0.129
Current drinker G3	119	2	2,119	0.9 (0.2–3.8)	0.60 (0.14–2.67)	0.52 (0.11–2.36)	0.790
Lower triglycerides (*n* = 1,863; <150 mg/dL)							
Non-drinker	670	34	11,343	3.0 (2.1–4.2)	Ref.	Ref.	
Past drinker	108	8	1,521	5.3 (2.6–10.5)	1.05 (0.48–2.28)	0.99 (0.45–2.14)	
Current drinker G1	572	21	9,876	2.1 (1.4–3.3)	0.80 (0.46–1.37)	0.70 (0.41–1.22)	
Current drinker G2	375	10	6,767	1.5 (0.8–2.7)	0.66 (0.32–1.34)	0.55 (0.27–1.13)	
Current drinker G3	138	3	2,510	1.2 (0.4–3.7)	0.88 (0.26–2.92)	0.69 (0.21–2.31)	

Lower HDL-C (*n* = 698; <40 mg/dL)							
Non-drinker	364	13	6,325	2.1 (1.2–3.5)	Ref.	Ref.	
Past drinker	62	6	944	6.4 (2.9–14.1)	1.80 (0.68–4.79)	2.00 (0.74–5.43)	0.275
Current drinker G1	157	7	2,754	2.5 (1.2–5.3)	1.25 (0.50–3.15)	1.21 (0.48–3.05)	0.072
Current drinker G2	75	2	1,370	1.5 (0.4–5.8)	1.23 (0.27–5.51)	1.21 (0.26–5.52)	0.367
Current drinker G3	40	1	706	1.4 (0.2–10.1)	1.08 (0.14–8.34)	1.04 (0.13–8.11)	0.459
Higher HDL-C (*n* = 2,211; ≥40 mg/dL)							
Non-drinker	647	34	11,090	3.1 (2.2–4.3)	Ref.	Ref.	
Past drinker	114	8	1,683	4.8 (2.4–9.5)	1.10 (0.51–2.38)	0.89 (0.41–1.95)	
Current drinker G1	707	19	12,444	1.5 (1.0–2.4)	0.56 (0.32–0.99)	0.46 (0.26–0.81)	
Current drinker G2	526	16	9,543	1.7 (1.0–2.7)	0.76 (0.42–1.38)	0.59 (0.32–1.10)	
Current drinker G3	217	4	3,923	1.0 (0.4–2.7)	0.60 (0.21–1.71)	0.44 (0.15–1.28)	

ASCVD, atherosclerotic cardiovascular disease; HDL-C, high-density lipoprotein cholesterol; py, person-years; HR, hazard ratio; CI, confidence interval

*per 1,000 py

**Adjusted for age, body mass index, diabetes, hypertension, current smoking, and exercise habit

Current drinkers G1 were defined as those who drank 3 or more days per week and about 1 gou of alcohol per day, G2 as those who drank 3 or more days per week and about 2 gou of alcohol per day, and G3 as those who drank 3 or more days per week and 3 gou or more of alcohol per day.

## Discussion

We investigated the association between alcohol consumption and death due to CVD using data from a long-term epidemiological study of Japanese men in the present study. The results showed that light drinking was significantly associated with lower risk of ASCVD, while moderate to heavy drinking was significantly associated with higher risk of cerebral hemorrhage. In addition, stratified analysis showed that light drinking was associated with lower risk of total CVD and ASCVD in participants without dyslipidemia or those with higher level of HDL-C. Our findings supported the benefit of small amounts of alcohol consumption for primary CVD prevention in Japanese men.

We showed that small amounts of alcohol consumption were significantly associated with lower risk of ASCVD among Japanese men in the present study. In the Japan collaborative cohort (JACC) study and the Japan public health center-based prospective (JPHC) study, low-to-moderate alcohol consumption was associated with lower risk of total CVD among Japanese men [9, 11]. Our findings were consistent with these studies, and we suggest that small amounts of alcohol consumption may be beneficial for primary ASCVD prevention in Japanese men. Several possible reasons might be proposed to explain the preventive effect of low-to-moderate alcohol consumption on death from ASCVD, which is shown in the present study. The major reason is that high alcohol consumption increases HDL-C levels, improve HDL-C function by promoting reverse cholesterol transport and decrease LDL-C levels, all of which prevent the progression of atherosclerosis. [13, 17, 30–32]. Other possible reason is owing to enhance insulin sensitivity, improve systemic inflammation, and reduce platelet aggregation by low-to-moderate alcohol consumption, which may also be beneficial to atherosclerosis [33–36].

We also showed that the protective effect of small amounts of alcohol consumption was observed in participants without dyslipidemia or participants with higher level of HDL-C only, who were expected to have a lower risk of atherosclerotic diseases. We first showed the impact of dyslipidemia on the association between alcohol consumption and CVD, and our findings suggest that current alcohol drinkers with dyslipidemia could not receive advantages of alcohol consumption for atherosclerosis, whereas current alcohol drinkers without dyslipidemia might receive advantages. One possible reason is that the preventive effect of alcohol consumption on atherosclerosis may not overcome the promoting effect of dyslipidemia on atherosclerosis. Other possible reason is that HDL-related gene polymorphisms which influenced levels of HDL-C by alcohol drinking, especially the polymorphisms of cholesteryl ester transfer protein (CETP) TaqIB gene, had protective effect on the ASCVD [37–40]. Further studies were warranted to clarify the mechanism that dyslipidemia or higher levels of HDL-C modified the protective effect of small amounts of alcohol consumption on atherosclerosis.

Another important finding is that excessive alcohol drinking was significantly associated with increasing risk of cerebral hemorrhage. In the JACC study and the JPHC study, large amounts of alcohol consumption were associated with increased risk of cerebral hemorrhage among Japanese men [5, 9, 11]. Also, in several studies of non-Japanese population, excessive alcohol drinking is associated with increased risk of cerebral hemorrhage [41–43]. Our findings were consistent with results of these studies. The estimated major mechanism for developing cerebral hemorrhage is due to elevated blood pressure or developed hypertension, which was induced by excessive alcohol consumption [43, 44]. We suggest that it may be important to control blood pressure as well as the reduction of alcohol consumption to prevent the incidence of cerebral hemorrhage in heavy alcohol drinkers.

The present study has several limitations. First, we did not measure the amount of alcohol consumed per day accurately because we measured the amount of alcohol consumed per day using the questionnaire, which cannot measure data on the frequency of drinking accurately. However, γ-GTP levels increased with the amount of alcohol consumption, which suggests that the drinking category classification in this study may reflect actual alcohol consumption accurately on a relative level. Second, the number of deaths from CVD or its subtypes was small, which may have resulted in insufficient power to detect the association between alcohol consumption and each cause-specific death. Finally, the present study was conducted only in men because of the small number of deaths and heavy drinkers in women. Thus, the results of the present study cannot be applied to women.

In conclusion, we showed that drinking one *gou* of alcohol per day was significantly associated with lower risk of CVD, especially ASCVD, in Japanese men. These results suggest that small amounts of alcohol may be protective against the risk of ASCVD. Especially, small amounts of alcohol reduced risk of ASCVD in participants without dyslipidemia or those with higher level of HDL-C. On the other hand, the risk of death from cerebral hemorrhage was significantly increased in participants with excessive current drinkers. Therefore, we suggest that it is important for current Japanese drinkers to limit their drinking to small amounts of alcohol, to reduce the risk of ASCVD.

## Supplementary information

**Additional file 1:** **Supplemental table 1:** Associations between alcohol consumption (continuous) and cardiovascular disease death, atherosclerotic cardiovascular disease death, and coronary heart disease death in men. **Supplemental table 2:** Associations between alcohol consumption (continuous) and ischemic stroke death and cerebral hemorrhage death in men.

## Declarations

### Availability of data and material

Data could not be provided for ethical reasons.
