# Supplementary material for: Association between alcohol intake and death from cardiovascular diseases and its subtypes stratified by dyslipidemia in Japanese men: 20-years follow-up of NIPPON DATA90
Source: Environ Health Prev Med. 2024 Nov 2;29:61. doi: 10.1265/ehpm.24-00164 (PMC11551442; doi:10.1265/ehpm.24-00164)
Supplement: Supplementary file 1 — Additional file 1: Supplemental table 1: Associations between alcohol consumption (continuous) and cardiovascular disease death, atherosclerotic cardiovascular disease death, and coronary heart disease death in men. Supplemental table 2: Associations between alcohol consumption (continuous) and ischemic stroke death and cerebral hemorrhage death in men. [file ehpm-29-061-s001.docx]

| Supplemental table 1: Associations between alcohol consumption (continuous) and cardiovascular disease death, Atherosclerotic cardiovascular disease death, and coronary heart disease death in men | | | | |
| --- | --- | --- | --- | --- |
|  | Age-adjusted HR (95% CI) | p-value | Multivariable-adjusted HR* 　 (95% CI) | p-value |
| Total cardiovascular disease |  |  |  |  |
| Overall (n=2,733) | 0.99 (0.85-1.15) | 0.869 | 0.91 (0.78-1.07) | 0.276 |
|  |  |  |  |  |
| With dyslipidemia (n=1,411) | 1.05 (0.84-1.30) | 0.673 | 1.03 (0.82-1.28) | 0.811 |
| Without dyslipidemia (n=1,322) | 0.94 (0.75-1.17) | 0.575 | 0.81 (0.64-1.03) | 0.081 |
|  |  |  |  |  |
| Higher non-HDL-C (n = 720; ≥170 mg/dL) | 1.21 (0.90-1.64) | 0.204 | 1.16 (0.85-1.59) | 0.342 |
| Lower non-HDL-C (n = 2,013; <170 mg/dL) | 0.92 (0.77-1.11) | 0.394 | 0.85 (0.70-1.02) | 0.082 |
|  |  |  |  |  |
| Higher triglycerides (n = 978; ≥150 mg/dL) | 1.11 (0.86-1.44) | 0.408 | 1.05 (0.81-1.37) | 0.698 |
| Lower triglycerides (n = 1,755; <150 mg/dL) | 0.94 (0.77-1.15) | 0.536 | 0.86 (0.70-1.06) | 0.161 |
|  |  |  |  |  |
| Lower HDL-C (n = 636; <40 mg/dL) | 0.93 (0.64-1.33) | 0.677 | 0.91 (0.62-1.32) | 0.610 |
| Higher HDL-C (n = 2,097; ≥40 mg/dL) | 1.02 (0.85-1.22) | 0.837 | 0.92 (0.76-1.11) | 0.373 |
|  |  |  |  |  |
| Atherosclerotic cardiovascular disease |  |  |  |  |
| Overall (n=2,733) | 0.90 (0.71-1.13) | 0.355 | 0.80 (0.63-1.02) | 0.074 |
|  |  |  |  |  |
| With dyslipidemia (n=1,411) | 0.97 (0.70-1.33) | 0.832 | 0.94 (0.68-1.31) | 0.727 |
| Without dyslipidemia (n=1,322) | 0.84 (0.60-1.16) | 0.291 | 0.68 (0.48-0.97) | 0.035 |
|  |  |  |  |  |
| Higher non-HDL-C (n = 720; ≥170 mg/dL) | 0.98 (0.63-1.51) | 0.911 | 0.95 (0.60-1.50) | 0.816 |
| Lower non-HDL-C (n = 2,013; <170 mg/dL) | 0.89 (0.68-1.16) | 0.378 | 0.78 (0.59-1.03) | 0.085 |
|  |  |  |  |  |
| Higher triglycerides (n = 978; ≥150 mg/dL) | 0.98 (0.67-1.44) | 0.932 | 0.90 (0.61-1.35) | 0.621 |
| Lower triglycerides (n = 1,755; <150 mg/dL) | 0.87 (0.65-1.16) | 0.345 | 0.79 (0.59-1.06) | 0.119 |
|  |  |  |  |  |
| Lower HDL-C (n = 636; <40 mg/dL) | 1.11 (0.68-1.80) | 0.674 | 1.09 (0.66-1.79) | 0.739 |
| Higher HDL-C (n = 2,097; ≥40 mg/dL) | 0.85 (0.65-1.11) | 0.233 | 0.75 (0.57-0.995) | 0.046 |
|  |  |  |  |  |
| Coronary heart disease |  |  |  |  |
| Overall (n=2,733) | 0.91 (0.66-1.24) | 0.534 | 0.82 (0.59-1.14) | 0.235 |
|  |  |  |  |  |
| With dyslipidemia (n=1,411) | 0.91 (0.60-1.39) | 0.678 | 0.85 (0.55-1.30) | 0.450 |
| Without dyslipidemia (n=1,322) | 0.91 (0.57-1.46) | 0.697 | 0.77 (0.47-1.28) | 0.317 |
|  |  |  |  |  |
| Higher non-HDL-C (n = 720; ≥170 mg/dL) | 0.92 (0.53-1.60) | 0.767 | 0.88 (0.50-1.56) | 0.662 |
| Lower non-HDL-C (n = 2,013; <170 mg/dL) | 0.93 (0.64-1.35) | 0.699 | 0.79 (0.53-1.18) | 0.254 |
|  |  |  |  |  |
| Higher triglycerides (n = 978; ≥150 mg/dL) | 0.90 (0.53-1.54) | 0.707 | 0.77 (0.44-1.35) | 0.367 |
| Lower triglycerides (n = 1,755; <150 mg/dL) | 0.92 (0.62-1.36) | 0.667 | 0.81 (0.54-1.22) | 0.317 |
|  |  |  |  |  |
| Lower HDL-C (n = 636; <40 mg/dL) | 1.05 (0.57-1.91) | 0.880 | 0.95 (0.50-1.78) | 0.866 |
| Higher HDL-C (n = 2,097; ≥40 mg/dL) | 0.91 (0.63-1.32) | 0.630 | 0.85 (0.58-1.26) | 0.419 |
| HR, hazard ratio; CI, confidence interval |  |  |  |  |
| *Adjusted for age, body mass index, diabetes, hypertension, current smoking, exercise habit and dyslipidemia | | | | |

| Supplemental table 2: Associations between alcohol consumption (continuous) and ischemic stroke death and cerebral hemorrhage death in men | | | | |
| --- | --- | --- | --- | --- |
|  | Age-adjusted HR (95% CI) | p-value | Multivariable-adjusted HR* 　 (95% CI) | p-value |
| Ischemic Stroke |  |  |  |  |
| Overall (n=2,733) | 0.89 (0.63-1.24) | 0.476 | 0.77 (0.54-1.11) | 0.161 |
|  |  |  |  |  |
| With dyslipidemia (n=1,411) | 1.04 (0.64-1.68) | 0.872 | 1.12 (0.67-1.87) | 0.669 |
| Without dyslipidemia (n=1,322) | 0.77 (0.48-1.24) | 0.285 | 0.61 (0.37-1.02) | 0.059 |
|  |  |  |  |  |
| Higher non-HDL-C (n = 720; ≥170 mg/dL) | 1.13 (0.57-2.25) | 0.732 | 1.18 (0.53-2.60) | 0.682 |
| Lower non-HDL-C (n = 2,013; <170 mg/dL) | 0.84 (0.57-1.23) | 0.373 | 0.76 (0.51-1.12) | 0.168 |
|  |  |  |  |  |
| Higher triglycerides (n = 978; ≥150 mg/dL) | 1.06 (0.62-1.80) | 0.841 | 1.10 (0.62-1.96) | 0.748 |
| Lower triglycerides (n = 1,755; <150 mg/dL) | 0.82 (0.53-1.27) | 0.373 | 0.76 (0.49-1.18) | 0.222 |
|  |  |  |  |  |
| Lower HDL-C (n = 636; <40 mg/dL) | 1.15 (0.51-2.62) | 0.739 | 1.45 (0.59-3.56) | 0.416 |
| Higher HDL-C (n = 2,097; ≥40 mg/dL) | 0.80 (0.55-1.16) | 0.236 | 0.66 (0.44-0.99) | 0.046 |
|  |  |  |  |  |
| Cerebral hemorrhage |  |  |  |  |
| Overall (n=2,733) | 1.74 (1.16-2.62) | 0.008 | 1.58 (1.03-2.44) | 0.036 |
|  |  |  |  |  |
| With dyslipidemia (n=1,411) | 1.99 (1.09-3.61) | 0.024 | 1.80 (0.97-3.36) | 0.062 |
| Without dyslipidemia (n=1,322) | 1.63 (0.90-2.94) | 0.105 | 1.45 (0.78-2.70) | 0.243 |
|  |  |  |  |  |
| Higher non-HDL-C (n = 720; ≥170 mg/dL) | 3.43 (1.29-9.13) | 0.014 | 3.23 (1.16-8.98) | 0.025 |
| Lower non-HDL-C (n = 2,013; <170 mg/dL) | 1.45 (0.91-2.33) | 0.121 | 1.34 (0.81-2.21) | 0.249 |
|  |  |  |  |  |
| Higher triglycerides (n = 978; ≥150 mg/dL) | 2.39 (1.17-4.88) | 0.017 | 2.08 (0.999-4.34) | 0.050 |
| Lower triglycerides (n = 1,755; <150 mg/dL) | 1.50 (0.88-2.54) | 0.137 | 1.37 (0.79-2.39) | 0.261 |
|  |  |  |  |  |
| Lower HDL-C (n = 636; <40 mg/dL) | 0.46 (0.08-2.74) | 0.391 | 0.36 (0.05-2.37) | 0.287 |
| Higher HDL-C (n = 2,097; ≥40 mg/dL) | 2.18 (1.35-3.51) | 0.001 | 2.03 (1.23-3.36) | 0.006 |
| HR, hazard ratio; CI, confidence interval |  |  |  |  |
| *Adjusted for age, body mass index, diabetes, hypertension, current smoking, exercise habit and dyslipidemia | | | | |
